# Developmental variation in resting-state EEG spectral correlates of response inhibition in children and adolescents

**DOI:** 10.1016/j.dcn.2026.101834

**Published:** 2026-10-06

**Authors:** Yanxi Xu, Ziqi Yang, Gaoxiang Ouyang, Qinghua He, Mengsi Xu

**Affiliations:** a School of Psychology, Shaanxi Normal University, Xi’an, China; b Department of Basic Psychology, Faculty of Medical Psychology, Army Medical University (Third Military Medical University), Chongqing, China; c State Key Laboratory of Cognitive Neuroscience and Learning, Beijing Normal University, Beijing, China; d Faculty of Psychology, Southwest University, Chongqing, China

**Keywords:** Response inhibition, Resting-state EEG, Spectral power, Band-ratio indices, Developmental variation

## Abstract

Identifying electrophysiological markers of response inhibition in children and adolescents is of substantial importance. Although previous studies have examined associations between resting-state electroencephalography (rs-EEG) power and response inhibition, findings across frequency bands have been inconsistent, and evidence regarding band-ratio indices remains limited. To address these issues, the present study examined the associations between rs-EEG spectral indices and response inhibition in a large sample of 1654 children and adolescents aged 6–18 years, and further tested whether these associations varied as a function of age. Based on eyes-open rs-EEG recordings, relative power was extracted for the delta, theta, alpha, and beta bands, and three band-ratio indices were calculated: the theta/beta ratio (TBR), alpha/theta ratio (ATR), and beta/theta ratio (BTR). Response inhibition was assessed using the stop-signal task. Correlation analyses showed that higher delta power, theta power, and TBR were associated with poorer response inhibition, whereas higher alpha power, beta power, ATR, and BTR were associated with better response inhibition. Moderation analyses further indicated that age significantly moderated the associations of delta power, beta power, ATR, and BTR with response inhibition, whereas no significant age-moderating effects were observed for theta power, alpha power or TBR. Taken together, these findings suggest that the electrophysiological correlates of response inhibition are developmentally dynamic across childhood and adolescence. The present study provides a psychophysiological account of developmental variation in response inhibition and underscores the importance of age when interpreting resting-state EEG-behavior associations.

## 1 Introduction

Response inhibition refers to the capacity to suppress inappropriate, impulsive, or prepotent motor responses in the service of goal-directed behavior, and is widely regarded as a core component of executive functioning (Diamond, 2013). During childhood and adolescence, the development of this capacity not only reflects broader cognitive maturation, but also predicts important functional outcomes, including academic achievement, social competence, and mental health (Moffitt et al., 2011). Conversely, poorer response inhibition during this developmental period has been associated with academic difficulties, poorer social adjustment, and a higher risk of externalizing problems, including Attention-Deficit/Hyperactivity Disorder (ADHD) (van Hulst et al., 2018, Xu et al., 2025). Accordingly, identifying neural correlates of response inhibition, and examining whether their behavioral significance varies across age, may help advance understanding of individual differences in response inhibition during childhood and adolescence.

Resting-state electroencephalography (rs-EEG) records spontaneous synchronous neuronal activity in the absence of explicit task demands and is widely used to assess baseline brain function and developmental status (Deco et al., 2011). Therefore, investigating the association between response inhibition and resting-state EEG can provide deeper insights into the neural processes associated with response inhibition that are not influenced by external task demands. Power spectral analysis is a central approach for characterizing rs-EEG signals, as it quantifies the distribution of neural activity across different frequency bands (Cole and Voytek, 2017, Newson and Thiagarajan, 2019). On this basis, ongoing neural oscillations are commonly divided into four physiologically meaningful bands: delta (1–4 Hz), theta (4–8 Hz), alpha (8–13 Hz), and beta (13–30 Hz) (Cole and Voytek, 2017). Previous research suggests that these frequency bands may be differentially related to inhibitory and control-related processes. In particular, theta activity has often been linked to cognitive control, conflict monitoring, and control implementation (Cavanagh and Frank, 2014); alpha activity has been associated with functional inhibition and the selective gating of task-relevant information (Klimesch, 2012); and beta activity has been related to the maintenance of the current cognitive or sensorimotor state (Engel and Fries, 2010). By contrast, the functional significance of delta activity in higher-order cognition remains less clear, although some accounts have suggested that sustained delta oscillations may contribute to the suppression of task-irrelevant interference (Harmony, 2013). Taken together, these findings suggest that rs-EEG spectral activity may provide useful information for understanding the neural basis of response inhibition during childhood and adolescence.

Although a growing number of studies have examined the associations between rs-EEG power and response inhibition in developmental samples, the available evidence remains inconclusive. Previous findings differ not only in the frequency bands implicated but also in the direction of the reported associations. One possible explanation for these inconsistencies is that prior studies have examined samples spanning different age ranges, even though childhood and adolescence constitute a critical developmental period during which both response inhibition and neural activity undergo substantial change (Anderson and Perone, 2018, Luna et al., 2004, Miskovic et al., 2015). From a developmental perspective, lower-frequency activity, particularly delta and theta power, generally decreases with age, whereas relatively higher-frequency activity, including alpha and beta power, tends to increase (Miskovic et al., 2015). This process of ‘spectral reorganization’ has been proposed to reflect a developmental transition from relatively diffuse, long-range neural synchrony toward more localized and refined neural regulation (Uhlhaas et al., 2010). Because both inhibitory performance and EEG spectral characteristics change across development, their associations may not remain constant but may instead vary systematically as a function of maturational stage.

Preliminary evidence for such age-dependent associations has been reported for theta activity. Pscherer et al. (2021) examined whether the association between resting-state theta power and response inhibition varied with age in healthy individuals aged 8–30 years. Using eyes-open rs-EEG and a Go/NoGo task, they found that, although higher theta power was associated with poorer response inhibition in the sample as a whole, this association varied as a function of age. Specifically, the association was not significant at younger ages, whereas after approximately 19.5 years of age, higher theta power was associated with better response inhibition. These findings provide preliminary evidence that age may moderate the association between rs-EEG power and response inhibition. However, that study examined only one frequency band, included participants extending into early adulthood rather than focusing specifically on childhood and adolescence, and used a relatively modest sample for detecting developmental moderation effects (*N* = 166). Potential developmental differences have also emerged in studies of other frequency bands. For delta activity, Aldemir et al. (2018) found that children with attention-deficit/hyperactivity disorder (ADHD) aged 7–12 years exhibited higher delta power than typically developing children, tentatively suggesting that elevated delta activity may be associated with weaker inhibitory functioning during childhood. In contrast, Dong et al. (2021) reported that, among young adults aged 18–23 years, greater delta power was associated with fewer NoGo errors, suggesting that higher delta activity may be related to better response inhibition at a later developmental stage. Findings for theta power are similarly mixed. A developmental review by Tan et al. (2024) concluded that higher theta power is generally associated with poorer inhibitory performance, whereas Pscherer et al. (2021) found no stable association between theta power and response inhibition during childhood and adolescence. In the relatively higher-frequency bands, Whedon et al. (2020) reported in a longitudinal study that greater increases in alpha power from 10 months to 4 years of age predicted better response inhibition at age 4. By contrast, Zhang et al. (2026) found that higher alpha power was associated with poorer response inhibition among children aged 6–12 years. Taken together, these findings suggest that associations between single-band power and response inhibition may depend on developmental stage. The same frequency band may have different implications for behavioral performance across age periods, depending on its position within the developmental trajectory of spectral reorganization. Nevertheless, beyond the preliminary investigation by Pscherer et al. (2021), research has not systematically examined whether associations between multiple rs-EEG frequency-band measures and response inhibition vary with age, particularly across childhood and adolescence.

In addition to single-band power, band-ratio indices may offer complementary information about developmental differences in EEG spectral organization. These indices are calculated by expressing the power of one frequency band relative to that of another, with commonly examined measures including the theta/beta ratio (TBR), alpha/theta ratio (ATR), and beta/theta ratio (BTR). Unlike measures of absolute or relative power within an individual band, band-ratio indices are generally interpreted as reflecting the relative dominance or balance of different spectral components (Anderson and Perone, 2018, Arns et al., 2013, Donoghue et al., 2020, Tan et al., 2024). Because childhood and adolescence are characterized by a gradual reduction in lower-frequency activity and a relative increase in higher-frequency activity, band-ratio indices may be particularly sensitive to developmental variation in spectral organization. Their inclusion may therefore provide a complementary perspective on the relationship between rs-EEG activity and response inhibition. However, evidence concerning associations between band-ratio indices and response inhibition remains limited. Among these indices, TBR has received the most attention, particularly in studies of children with ADHD. For example, Lin et al. (2021) recorded rs-EEG in children with ADHD aged 8–12 years and assessed interference control using a flanker task. Higher TBR was associated with poorer interference-control performance. Evidence regarding ATR and BTR is even more limited. To our knowledge, Cai et al. (2021) conducted one of the few studies to examine these indices in children aged 7–9 years, using the Receptive Attention Task as a measure of inhibitory performance. Both ATR and BTR positively predicted task performance. However, the task did not directly assess response inhibition, and the study included only a relatively narrow age range. It therefore remains unclear whether ATR and BTR are associated specifically with response inhibition and whether such associations generalize across the broader developmental period spanning childhood and adolescence. Overall, previous research suggests that band-ratio indices may be associated with control-related abilities in children, but the evidence is limited in two important respects. First, prior studies have primarily examined attentional or interference control rather than response inhibition directly. Second, they have not tested whether age moderates associations between band-ratio indices and behavioral performance. This omission is theoretically important because EEG frequency bands undergo systematic developmental changes across childhood and adolescence (Anderson and Perone, 2018, Miskovic et al., 2015), and indices representing the relative balance between frequency bands may likewise have different behavioral implications across developmental stages. Examining only overall associations is therefore insufficient to determine whether band-ratio indices relate to response inhibition consistently across childhood and adolescence.

Taken together, existing research has not yet clarified whether the associations between rs-EEG spectral indices and response inhibition depend on developmental stage, or whether such developmental dependence is present for both single-band power and band-ratio indices. To address these questions, the present study examined the associations between rs-EEG spectral indices—including both single-band power and band ratio indices—and response inhibition in a large sample of children and adolescents aged 6–18 years, and further tested whether age moderated these EEG-behavior associations. Given the inconsistent findings concerning single-band power and the limited direct evidence regarding band-ratio indices, the present study did not advance specific directional hypotheses.

## 2 Method

### 2.1 Participants

This study used data from the Chinese Child Brain Development Cohort Study (CCBD project), a large-scale initiative designed to investigate brain and cognitive development in Chinese children and adolescents (Zhang and Xue, 2025). The current sample included 1654 participants, comprising 705 females, aged 6–18 years (M = 12.0, SD = 3.5). All participants reported no history of neurological or psychiatric disorders and had normal or corrected-to-normal vision. The study protocol was approved by the local Institutional Review Board. Written informed consent was obtained from all participants and their legal guardians prior to participation.

### 2.2 The stop-signal task

The stop-signal task was used to assess response inhibition (Verbruggen and Logan, 2008). In this task (Fig. 1), participants were instructed to press the left or right response key according to the direction of a left- or right-pointing arrow on trials without a stop signal (Go trials), and to withhold their response when a stop signal was presented (Stop trials).

**Fig. 1:**
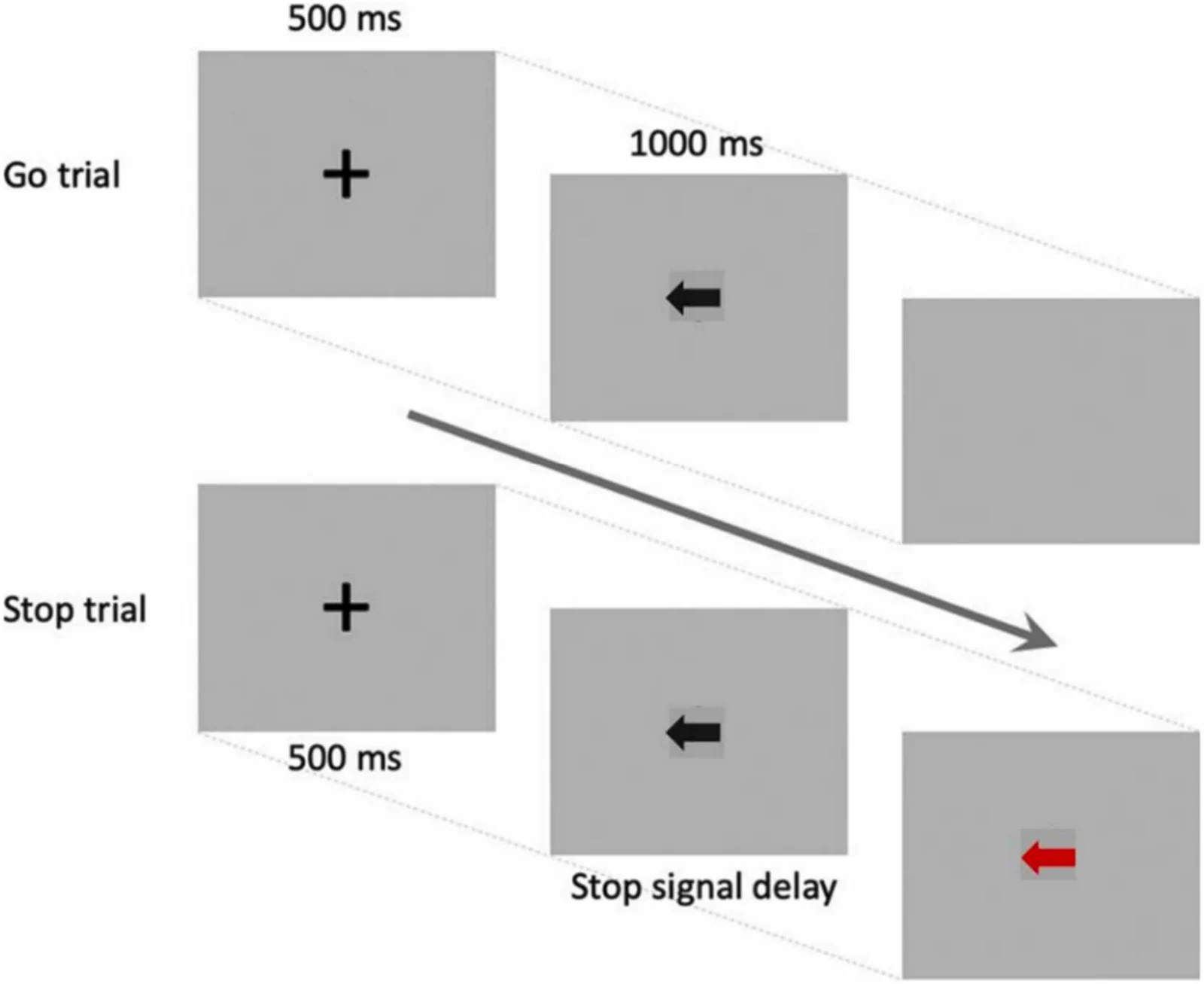
Illustrations of the stop-signal task.

Specifically, each trial began with a central fixation cross (“+”) presented for 500 ms, followed by a black arrow pointing either left or right for a maximum of 1000 ms. On Go trials, participants were required to indicate the direction of the arrow as quickly as possible by pressing the corresponding key. On Stop trials, the black arrow turned red after a variable stop-signal delay (SSD), which served as the stop signal and instructed participants to withhold their response. A standard staircase-tracking procedure was used to adjust SSD adaptively. The initial SSD was set to 200 ms. After each successfully inhibited Stop trial, SSD increased by 50 ms to make stopping more difficult; after each failed Stop trial, SSD decreased by 50 ms to make stopping easier. Following a response, or after the stimulus reached its maximum presentation time, the arrow disappeared and accuracy feedback was presented for 500 ms. To ensure that participants fully understood the task instructions, all participants first completed a 16-trial practice block and were required to achieve an overall accuracy of at least 70% before proceeding to the formal experiment. The formal task consisted of 256 trials divided into four blocks, including 75% Go trials and 25% Stop trials. Throughout the task, participants were instructed to respond as quickly and accurately as possible.

Stop-signal reaction time (SSRT) was treated as the primary behavioral index of response inhibition. Because SSRT cannot be directly observed in the stop-signal task, it must be estimated indirectly. On Stop trials, only the behavioral outcome—successful inhibition or, in the case of failed stopping, an overt response—can be observed, whereas the latent duration of the stopping process itself cannot be measured directly. Accordingly, unlike reaction time (RT) on Go trials, SSRT was estimated on the basis of the independent race model (Logan and Cowan, 1984) using the integration method recommended by Verbruggen et al. (2019). Specifically, the overall probability of responding on Stop trials, p (respond | stop), was first calculated. All Go trials with a response were then rank-ordered from fastest to slowest, and Go omissions were assigned an RT of 1000 ms, corresponding to the maximum stimulus duration. The RT at the percentile corresponding to p (respond | stop) in the ordered Go RT distribution was taken as an estimate of the finishing time of the stop process, and mean SSD was subtracted from this value to obtain SSRT. Mean Go RT was defined as the average RT across correct Go trials. A shorter SSRT was interpreted as indicating less time required to complete the stopping process and, therefore, stronger response inhibition.

### 2.3 Experimental procedure

After the EEG cap was fitted, participants first completed the resting-state EEG recording. During the recording, they were seated approximately 1 m from the computer screen and instructed to look directly at a central fixation cross (‘+’) with their eyes open. Verbal instructions were provided before the recording began, and an auditory cue indicated the end of the recording period. Participants were instructed to sit comfortably, remain relaxed, minimize movement, and remain quiet throughout the recording. EEG was recorded only during this resting-state period and was not collected during the subsequent stop-signal task.

Following the resting-state EEG recording, participants completed a practice block of the stop-signal task before proceeding to the formal task. As described above, an overall accuracy of at least 70% in the practice block was required to ensure adequate understanding of the task instructions. Participants who did not meet this criterion were permitted to repeat the practice block, for a maximum of five attempts. Those who failed to achieve the 70% accuracy threshold after five attempts were excluded from further participation.

### 2.4 EEG recordings and analysis

Resting-state EEG data for the present study were collected across multiple CCBD acquisition sites, including Beijing Normal University, Central China Normal University, East China Normal University, Guangzhou Women and Children’s Medical Center, Guizhou Normal University, Henan Normal University, Hangzhou Normal University, Liaoning Normal University, Northeast Normal University, Peking University, the Institute of Psychology, Chinese Academy of Sciences, Shandong Normal University, Southeast University, ShanghaiTech University, Sichuan Normal University, Shenzhen Institute of Neuroscience, Shaanxi Normal University, Southwest University, Tianjin Normal University, the Second Xiangya Hospital of Central South University, Zhejiang University, and Henan Children’s Hospital/Zhengzhou Children’s Hospital. EEG signals were continuously recorded using a 64-channel EGI system (Electrical Geodesics, Inc., Eugene, OR, USA), with CPz serving as the online reference. Electrode impedances were kept below 50 kΩ, and the sampling rate was 1000 Hz. Data from four cheek electrodes, channels 61–64, were excluded from further analysis because these sites are particularly vulnerable to contamination from facial expressions and muscle activity. A 3-min continuous eyes-open resting-state recording was obtained for each participant and used for subsequent analyses.

EEG preprocessing and spectral feature extraction were performed centrally by the CCBD core data-processing team with a standardized pipeline in MATLAB (The MathWorks, Natick, MA, USA) using custom scripts in combination with the EEGLAB toolbox (Delorme and Makeig, 2004). To objectively assess data quality, bad epochs in the raw EEG data were identified using a standardized automated quality-control algorithm (Zhao et al., 2023). An epoch was classified as bad if it met any of the following criteria: (1) constant or absent signal, defined as no detectable variation in signal amplitude within the epoch; (2) excessive amplitude, defined as an amplitude exceeding ±100 μV in any channel; (3) excessive high-frequency noise, defined as a Z score greater than 4 for the high-frequency (>40 Hz) noise-to-signal ratio; or (4) low spatial correlation, defined as a mean interchannel correlation coefficient below 0.40 within the epoch. A channel was classified as bad if more than 60% of its epochs were identified as bad. The continuous EEG data were then band-pass filtered from 1 to 40 Hz. Subsequently, bad channels were interpolated using the Reference Electrode Standardization Interpolation Technique (RESIT; Dong et al., 2021), and the data were rereferenced using the Reference Electrode Standardization Technique (REST; Yao, 2001). After a second round of data-quality inspection to identify any remaining artifact-contaminated segments, the continuous eyes-open resting-state data were segmented into 5-s epochs. Epochs containing marked artifacts were automatically excluded from subsequent spectral analyses. Eye-movement artifacts were further identified and removed using the ICA-based ADJUST algorithm, that is, Automatic EEG Artifact Detection based on the Joint Use of Spatial and Temporal Features (Mognon et al., 2011). Regions of interest (ROIs) were defined based on previous studies implicating fronto-central regions in inhibitory control and related electrophysiological processes (Huster et al., 2013, Parkinson and Haggard, 2015, Xu et al., 2020). Specifically, the fronto-central ROI included F1, F2, Fz, FC1, FC2, FCz, and Cz.

For spectral analysis, power was extracted for the delta (1–4 Hz), theta (4–8 Hz), alpha (8–13 Hz), and beta (13–30 Hz) bands. Relative power was used in the present study because it reduces between-individual variability and provides a more comparable index of EEG activity across participants (Cohen, 2017). Fast Fourier Transform (FFT) was applied to each artifact-free epoch at the selected electrodes, and the resulting power spectra were averaged. Relative power for each frequency band was then calculated by dividing the power within that band by the total power in the 1–30 Hz range. On this basis, three band-ratio indices were further computed: theta/beta ratio (TBR), defined as theta relative power divided by beta relative power; alpha/theta ratio (ATR), defined as alpha relative power divided by theta relative power; and beta/theta ratio (BTR), defined as beta relative power divided by theta relative power. The ratio measures examined in the present study were limited to commonly used indices with established relevance to cognitive and attentional functioning. Delta activity (1–4 Hz) is strongly associated with sleep-related and homeostatic processes (Ünsal et al., 2024), whereas its functional interpretation during wakefulness is relatively broad and less specific to particular cognitive processes. Therefore, delta-based ratios were not included in the primary analyses. In this way, resting-state EEG characteristics were characterized not only in terms of the magnitude of activity within individual frequency bands, but also in terms of the relative balance between bands, thereby providing a complementary description of spectral organization.

### 2.5 Statistical analysis

All statistical analyses were conducted in R. As an initial step, Pearson correlation coefficients were calculated to describe the bivariate associations among age, sex (0 = male, 1 = female), SSRT, relative power in the delta, theta, alpha, and beta bands, and the band-ratio indices TBR, ATR, and BTR. To reduce the likelihood of Type I error arising from multiple comparisons, p values were adjusted using the false discovery rate (FDR) procedure (Benjamini and Hochberg, 1995).

To test whether age moderated the associations between rs-EEG spectral indices and response inhibition, separate linear moderation models were estimated for each spectral index. In each model, one spectral index served as the predictor, SSRT as the dependent variable, age as the continuous moderator, and sex as a covariate. Prior to analysis, all continuous variables were z-score standardized to place them on a common scale and facilitate interpretation of the regression coefficients. Because standardization centers each variable at zero, the coefficients of the lower-order terms in models containing interactions represent their conditional effects when the other interacting variable is at its mean. Each model included the main effects of the spectral index, age, and sex, together with the spectral index × age interaction. To examine potential nonlinear age-related effects, we compared the linear models against quadratic models additionally containing the squared spectral term (EEG²) and its interaction with age using nested likelihood-ratio tests (LRT), AIC, and BIC. The linear models consistently showed superior or comparable fit (see Supplementary Table S1), and were therefore retained as the primary analytical framework. For the moderation analyses, regression coefficients were estimated using ordinary least squares. Because the residual diagnostic plots indicated heteroscedasticity, primary statistical inference was based on HC3 heteroscedasticity-consistent standard errors, corresponding two-sided *p* values, and Wald 95% confidence intervals. Statistical significance was determined on the basis of the HC3 inference. Given the right-skewness of the residuals, bootstrap percentile 95% confidence intervals based on 5000 resamples were additionally computed for the focal coefficients (age, the spectral index, and their interaction) as a sensitivity analysis (Hayes, 2017). The lower and upper limits of these bootstrap confidence intervals are reported in Supplementary Table S3.

For models in which the interaction term was significant under the primary HC3 inference, simple-slope analyses were conducted to estimate the conditional association between the spectral index and SSRT at the 16th, 50th, and 84th percentiles of age. Johnson–Neyman analyses were subsequently performed to identify the age ranges over which the conditional association between the spectral index and SSRT was statistically significant (Hayes, 2017). Both the simple-slope tests and Johnson–Neyman analyses were conducted using the same HC3 variance–covariance matrix as the primary moderation analyses. To facilitate interpretation, the Johnson–Neyman regions of significance were transformed back into the original age scale and reported in years. These regions represent statistical boundaries within the fitted cross-sectional models and should not be interpreted as precise developmental thresholds.

## 3 Results

Correlation analyses showed that age was significantly negatively correlated with SSRT (*r* = -.61, *p* < .001), indicating that older participants tended to show shorter SSRTs (Fig. 2). Among the rs-EEG spectral indices, age was significantly negatively correlated with delta power (*r* = -.19, *p* < .001), theta power (*r* = -.32, *p* < .001), and TBR (*r* = -.43, *p* < .001), but significantly positively correlated with alpha power (*r* = .12, *p* < .001), beta power (*r* = .40, *p* < .001), ATR (*r* = .24, *p* < .001), and BTR (*r* = .43, *p* < .001). SSRT was also significantly associated with all rs-EEG spectral indices. Specifically, SSRT was positively correlated with delta power (*r* = .19, *p* < .001), theta power (*r* = .18, *p* < .001), and TBR (*r* = .29, *p* < .001), but negatively correlated with alpha power (*r* = -.17, *p* < .001), beta power (*r* = -.25, *p* < .001), ATR (*r* = -.21, *p* < .001), and BTR (*r* = -.26, *p* < .001). Sex was significantly negatively correlated with SSRT (*r* = -.08, *p* = .001). In addition, sex was significantly associated with several rs-EEG spectral indices: It was negatively correlated with delta power (*r* = -.13, *p* < .001), theta power (*r* = -.06, *p* = .019), and TBR (*r* = -.18, *p* < .001), but positively correlated with beta power (*r* = .22, *p* < .001) and BTR (*r* = .20, *p* < .001). Specifically, male participants had significantly longer SSRTs (*r* = −.08, *p* = .001) and higher delta power (*r* = −.13, *p* < .001), theta power (*r* = −.06, *p* = .019), and TBR (*r* = −.18, *p* < .001) than female participants. In contrast, female participants had significantly higher beta power (*r* = .22, *p* < .001) and BTR (*r* = .20, *p* < .001) than male participants. No significant sex differences were observed in alpha power (*r* = .03, *p* = .230) or ATR (*r* = .04, *p* = .130). Because sex was significantly related to both SSRT and several EEG indices, it was included as a covariate in the subsequent moderation analyses.

**Fig. 2:**
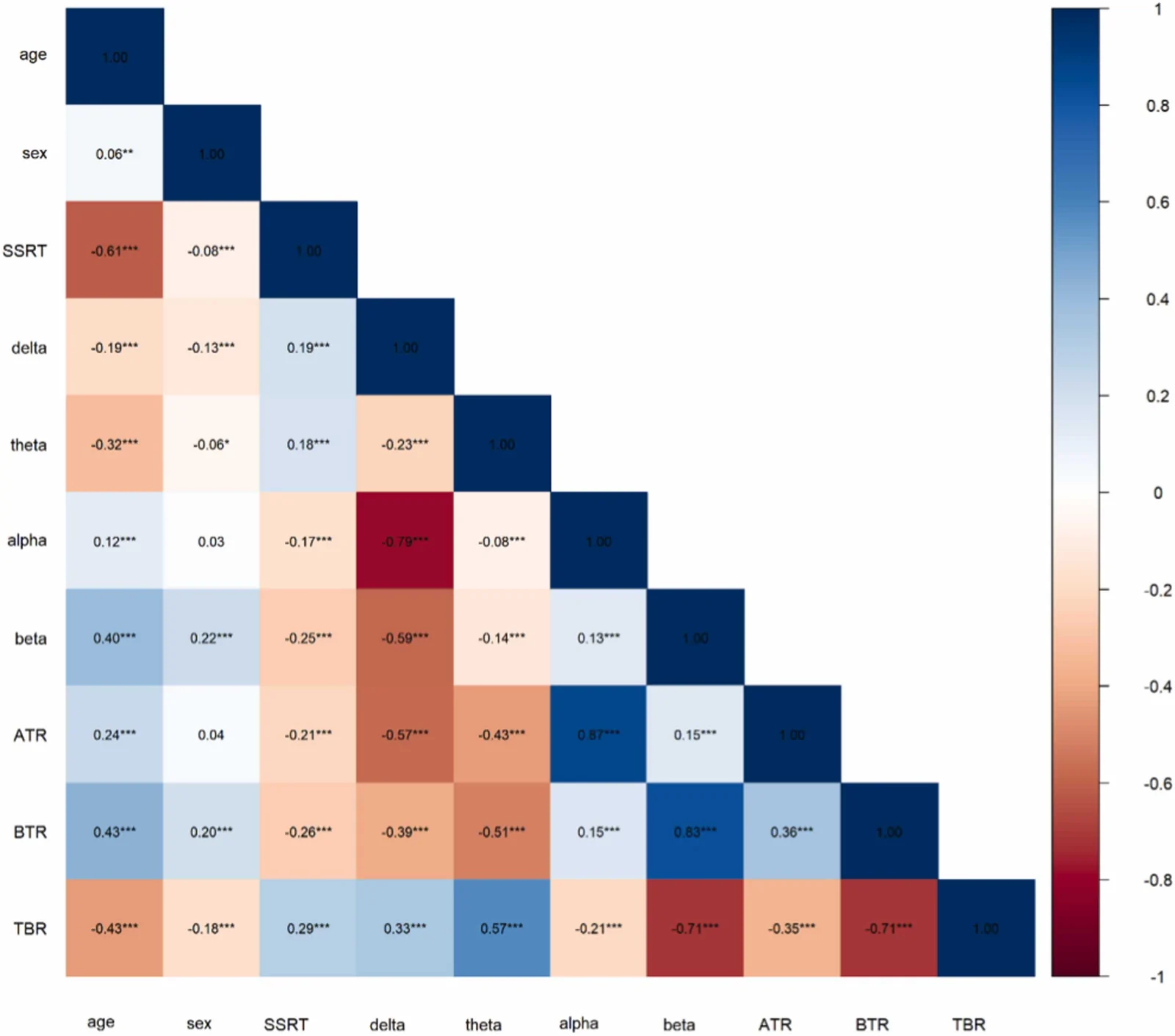
Bivariate correlations among age, sex, SSRT, and rs-EEG spectral indices (N = 1654).

Prior to testing the moderating role of age, we visualized the distributions of the spectral indices across ages 6–18 using scatterplots with LOESS-smoothed curves, stratified by sex (Supplementary Figure S8). As shown in Supplementary Figure S8, beta power and BTR generally increased with age, with the trends appearing to level off at approximately 14 years of age. These patterns were broadly comparable across males and females. The age-related distributions of the remaining spectral indices are also presented in Supplementary Figure S8. Importantly, these descriptive patterns are based on cross-sectional data and therefore reflect between-individual age differences rather than within-individual developmental trajectories. This descriptive overview provides a reference for the subsequent moderation analyses.

In the moderation analyses, age significantly moderated the associations of delta power, beta power, ATR, and BTR with SSRT, whereas no significant age-moderating effects were observed for theta power, alpha power and TBR (Table 1). For the single-band indices, in the delta model, both the main effect of delta power (β = 0.084, 95% CI [0.045, 0.123]) and the delta power × age interaction (β = −0.047, 95% CI [-0.087, −0.008]) were significant, in addition to the main effect of age (β = −0.592, 95% CI [-0.632, −0.551]). In the theta model, only the main effect of age was significant (β = −0.615, 95% CI [-0.658, −0.573]); neither the main effect of theta power (β = −0.019, 95% CI [-0.057, 0.019]) nor the theta power × age interaction (β = −0.025, 95% CI [-0.062, 0.012]) was significant. In the alpha model, both the main effect of age (β = −0.594, 95% CI [-0.634, −0.555]) and the main effect of alpha power (β = −0.102, 95% CI [-0.138, −0.066]) were significant, whereas the alpha power × age interaction was not (β = 0.028, 95% CI [-0.011, 0.067]). In the beta model, the main effect of age was significant (β = −0.600, 95% CI [-0.645, −0.555]) and the beta power × age interaction was also significant (β = 0.087, 95% CI [0.047, 0.128]), whereas the main effect of beta power was not (β = −0.031, 95% CI [-0.076, 0.014]). For the band-ratio indices, in the TBR model, the main effect of age was significant (β = −0.604, 95% CI [-0.649, −0.558]), whereas neither the main effect of TBR (β = 0.009, 95% CI [-0.037, 0.055]) nor the TBR × age interaction (β = −0.040, 95% CI [-0.086, 0.006]) was significant. In the ATR model, both the main effect of age (β = −0.579, 95% CI [-0.620, −0.538]) and the main effect of ATR (β = −0.111, 95% CI [-0.152, −0.069]) were significant, and the ATR × age interaction was also significant (β = 0.067, 95% CI [0.024, 0.110]). In the BTR model, the main effect of age was significant (β = −0.601, 95% CI [-0.649, −0.553]) and the BTR × age interaction was also significant (β = 0.092, 95% CI [0.041, 0.144]), whereas the main effect of BTR was not (β = −0.030, 95% CI [-0.089, 0.029]).

**Table 1:** Age as a moderator of the associations between rs-EEG spectral indices and SSRT, controlling for sex (*N* = 1654).

**Model term**	***β***	**Robust_SE**	**LLCI**	**ULCI**	***t***	***p***	***F***	***F_p***	***R***^***2***^	***R***^***2***^***_adj***	***∆R***^***2***^
**delta power**											
Age	-0.592	0.021	-0.632	-0.551	-28.688	< .001	276.405	< .001	0.384	0.383	0.002
delta	0.084	0.020	0.045	0.123	4.178	< .001					
Age × delta	-0.047	0.020	-0.087	-0.008	-2.355	0.019					
**theta power**											
Age	-0.615	0.022	-0.658	-0.573	-28.334	< .001	269.023	< .001	0.378	0.376	0.001
theta	-0.019	0.019	-0.057	0.019	-0.959	0.338					
Age × theta	-0.025	0.019	-0.062	0.012	-1.327	0.185					
**alpha power**											
Age	-0.594	0.020	-0.634	-0.555	-29.432	< .001	278.659	< .001	0.386	0.385	0.001
alpha	-0.102	0.018	-0.138	-0.066	-5.528	< .001					
Age × alpha	0.028	0.020	-0.011	0.067	1.418	0.156					
**beta power**											
Age	-0.600	0.023	-0.645	-0.555	-26.299	< .001	276.568	< .001	0.384	0.383	0.007
beta	-0.031	0.023	-0.076	0.014	-1.338	0.181					
Age × beta	0.087	0.021	0.047	0.128	4.215	< .001					
**ATR ratio**											
Age	-0.579	0.021	-0.620	-0.538	-27.951	< .001	277.186	< .001	0.385	0.383	0.003
ATR	-0.111	0.021	-0.152	-0.069	-5.219	< .001					
Age × ATR	0.067	0.022	0.024	0.110	3.066	0.002					
**BTR ratio**											
Age	-0.601	0.025	-0.649	-0.553	-24.495	< .001	276.595	< .001	0.384	0.383	0.007
BTR	-0.030	0.030	-0.089	0.029	-0.986	0.324					
Age × BTR	0.092	0.026	0.041	0.144	3.517	< .001					
**TBR ratio**											
Age	-0.604	0.023	-0.649	-0.558	-25.734	< .001	270.499	< .001	0.379	0.378	0.002
TBR	0.009	0.023	-0.037	0.055	0.370	0.712					
Age × TBR	-0.040	0.024	-0.086	0.006	-1.695	0.090					

Note: *β* = standardized regression coefficient; Robust_SE = HC3 heteroscedasticity-consistent standard error; LLCI and ULCI = lower and upper limits, respectively, of the HC3-based Wald 95% confidence interval; *F* = HC3-based omnibus Wald *F* statistic; *F_p* = associated *p* value; *R*²_adj = adjusted *R*²; Δ*R²* = increase in *R²* from the main-effects model (EEG + age + sex) to the interaction model (EEG + age + sex + EEG × age). All coefficient *p* values are two-sided.

Simple-slope analyses showed that the associations between the EEG spectral indices and SSRT differed across age levels. Among the indices, delta, beta, ATR and BTR showed significant interaction effects with age (Fig. 3). Specifically, delta power showed significant positive slopes at the 16th percentile of age (8 years) and the 50th percentile (11.5 years) (*B* = 0.134, *p* < .001; *B* = 0.087, *p* < .001), but a non-significant positive slope at the 84th percentile (16 years) (*B* = 0.026, *p* = .227), suggesting that its positive association with SSRT was primarily present in younger children. Beta power shifted from a significant negative association with SSRT in the younger group (*B* = −0.122, *p* = .002) to a non-significant association in the middle group (*B* = −0.036, *p* = .134), and then to a significant positive association in the older group (*B* = 0.076, *p* < .001). Similarly, ATR showed significant negative slopes in the younger (8 years: *B* = −0.181, *p* < .001) and middle (11.5 years: *B* = −0.114, *p* < .001) age groups, but a non-significant slope in the older group (16 years: *B* = −0.029, *p* = .132). BTR also changed from a negative association in the younger group (8 years: *B* = −0.127, *p* = .022) to a non-significant association in the middle group (11.5 years: *B* = −0.035, *p* = .268), and then to a positive association in the older group (16 years: *B* = 0.083, *p* < .001), closely mirroring the pattern observed for beta power. Overall, beta power and BTR exhibited a reversal from negative to positive associations, and the associations involving delta and ATR were more evident at younger ages, highlighting the age-dependent nature of the relationships between resting-state EEG spectral measures and response inhibition.

**Fig. 3:**
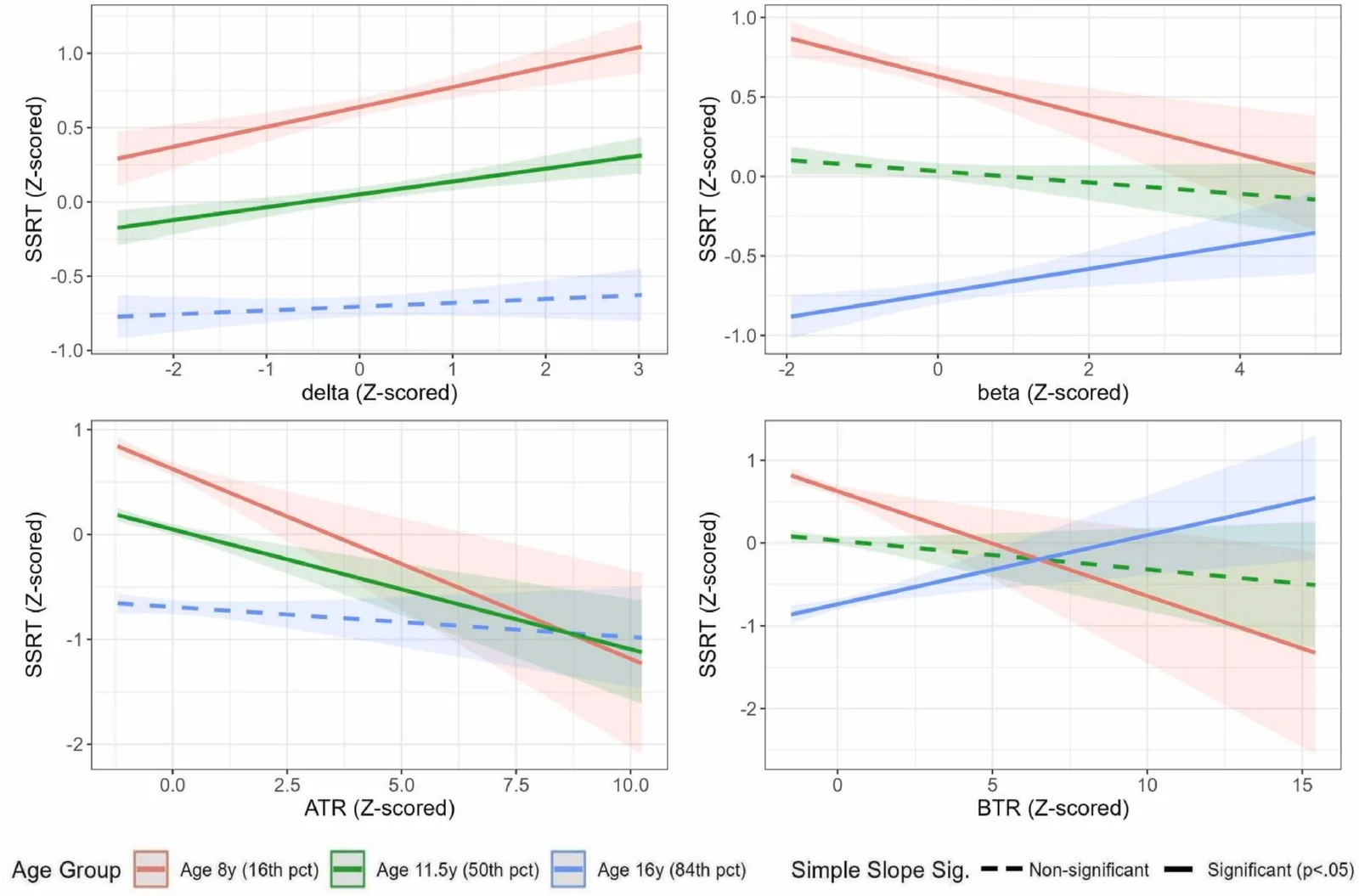
Simple-slope analyses for models showing significant rs-EEG spectral index × age interactions. Slopes are presented at the 16th, 50th, and 84th percentiles of age (8, 11.5, and 16 years, respectively). Solid lines indicate significant simple slopes (p < .05), whereas dashed lines indicate non-significant simple slopes.

Johnson–Neyman analyses were conducted for all significant interaction terms (Fig. 4). In the delta model, the positive conditional association between delta power and SSRT was significant only before 15.20 years of age. In the beta model, the conditional association between beta power and SSRT was significantly negative before 10.85 years of age, indicating that higher beta power was associated with shorter SSRTs, but became significantly positive after 14.47 years of age, indicating that higher beta power was associated with longer SSRTs; between 10.85 and 14.47 years of age, this association was not significant. In the ATR model, the negative conditional association between ATR and SSRT was significant only before 15.68 years of age and was no longer significant thereafter. In the BTR model, the pattern was similar to that observed for beta power: The conditional association between BTR and SSRT was significantly negative before 9.47 years of age, significantly positive after 14.17 years of age, and not significant between 9.47 and 14.17 years of age.

**Fig. 4:**
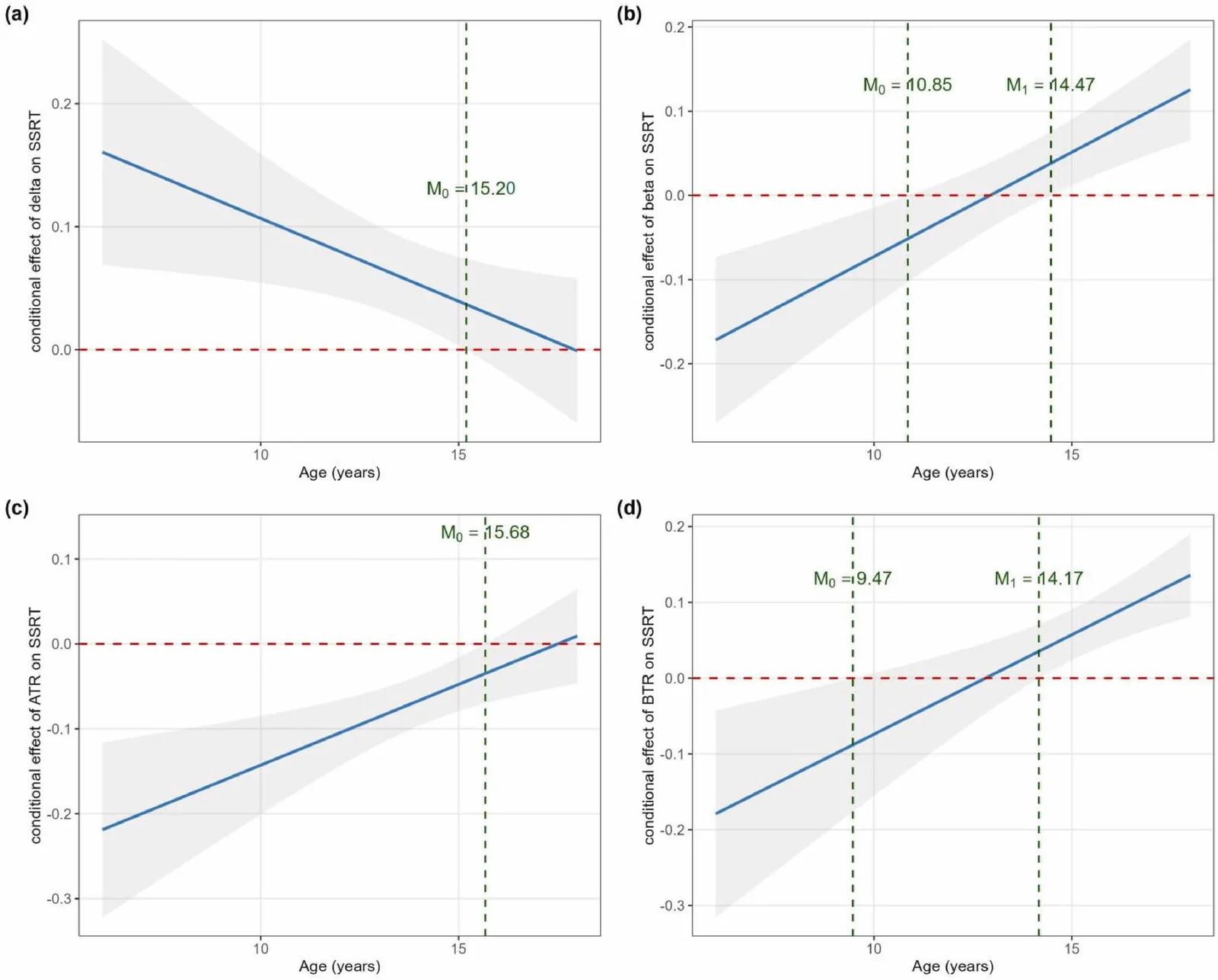
Johnson–Neyman plots showing the conditional effects of delta power, beta power, ATR and BTR on SSRT across age.

## 4 Discussion

The present study examined the associations between rs-EEG spectral indices and response inhibition in children and adolescents aged 6–18 years, and further tested whether these associations varied as a function of age. The results showed that several rs-EEG spectral indices were significantly associated with response inhibition. More importantly, age significantly moderated the associations of delta power, beta power, ATR, and BTR with SSRT, whereas the interactions involving theta power, alpha power, and TBR were not significant. These findings suggest that the relationship between rs-EEG spectral activity and response inhibition may be developmentally dynamic rather than fixed across childhood and adolescence. Although such age-related variation was not evident for all indices examined, it was observed for multiple spectral indicators, including both single-band and ratio-based measures. This pattern may indicate that developmental changes in response inhibition are reflected not only in specific frequency bands, but also in the relative organization of spectral components. Thus, the present findings may help to advance understanding of age-related variation in both response inhibition and rs-EEG spectral activity during childhood and adolescence.

The overall correlation analyses provide an important descriptive context for interpreting these findings. First, age was significantly associated with better response inhibition, as reflected by shorter SSRTs, suggesting that response inhibition improves across childhood and adolescence. This finding is consistent with previous developmental studies reporting age-related improvements in response inhibition (Albert et al., 2022, Bedard et al., 2002, Dimoska et al., 2007, Luna et al., 2004, Williams et al., 1999). Second, age was associated with a relatively typical pattern of EEG maturation: Indices related to low-frequency activity, including delta power, theta power, and TBR, decreased with age, whereas indices related to higher-frequency activity, including alpha power, beta power, ATR, and BTR, increased with age. Developmental changes in EEG spectral activity are closely related to neurobiological processes such as synaptic pruning, myelination, and the maturation of inhibitory neurotransmission, all of which contribute to the increasing efficiency and specialization of neural systems supporting cognitive control (Marek et al., 2018, Meng et al., 2022, Stanojević et al., 2023). During childhood, relatively high synaptic density and broadly distributed neural connectivity may contribute to stronger low-frequency activity and greater metabolic demands. With development, the elimination of redundant synaptic connections through synaptic pruning is thought to increase the efficiency and specialization of neural circuits, accompanied by reductions in low-frequency delta and theta activity (Uhlhaas et al., 2010). Consistent with this interpretation, developmental changes in EEG activity have been reported to correspond spatially and temporally with reductions in cortical gray matter (Segalowitz et al., 2010). The developmental pattern of higher-frequency activity is more complex. Although higher-frequency oscillations emerge early in life, the precise temporal coordination and large-scale synchronization of these oscillations continue to mature through adolescence and into early adulthood. This prolonged maturation is associated with ongoing myelination of corticocortical pathways and the development of GABAergic neurotransmission (Uhlhaas et al., 2010). Increasing myelination improves the speed and reliability of neural communication, whereas maturation of GABAergic inhibitory circuits supports more precise timing and synchronization of cortical activity. Together, these processes facilitate the emergence of more stable and efficient higher-frequency activity, including alpha and beta oscillations, which support executive functions such as attentional control, working memory, and response inhibition (Uhlhaas and Singer, 2010). From this perspective, resting-state spectral parameters may be particularly informative during childhood and adolescence because individuals differ substantially in the timing and extent of these ongoing maturational processes. Variability in low- and high-frequency activity may therefore reflect meaningful differences in the maturity and efficiency of neural systems supporting response inhibition. By later adolescence or early adulthood, however, synaptic organization, myelination, and inhibitory neurotransmission become relatively more stabilized. Consequently, resting spectral measures may show less developmentally driven variability and may account for less individual variation in response inhibition. This does not imply that these oscillations cease to contribute to inhibitory control in adults. Rather, their resting-state magnitude may become a less sensitive marker of individual differences in behavioral performance once the underlying neural systems have reached a more mature and stable level of organization. Third, higher delta power, theta power, and TBR were associated with poorer response inhibition, whereas higher alpha power, beta power, ATR, and BTR were associated with better response inhibition. Importantly, however, these overall correlations capture only the average linear associations across the full sample and do not indicate whether such associations are developmentally stable. This provided the rationale for the moderation analyses.

Before turning to the individual findings, it is important to distinguish between single-band power and band-ratio indices. Single-band power reflects activity within a specific frequency range (Cole and Voytek, 2017, Newson and Thiagarajan, 2019), whereas band-ratio indices characterize the relative balance between two frequency bands and may therefore be particularly sensitive to developmental variation in spectral organization (Donoghue et al., 2020, Finley et al., 2022). However, the present findings did not reveal a simple distinction between these two types of measures. Age significantly moderated the associations of delta and beta power, as well as ATR and BTR, with response inhibition, but not those of theta or alpha power. The TBR interaction was method-sensitive: It was not significant under the primary HC3-based inference, although its bootstrap percentile confidence interval excluded zero. Accordingly, the following findings are discussed by treating single-band power and band-ratio indices as related but non-interchangeable measures of resting-state spectral activity.

In the delta band, lower delta power was associated with better response inhibition overall, and this association was qualified by a significant interaction with age. Specifically, higher delta power was associated with poorer response inhibition only before 15.20 years of age. This pattern suggests that the relationship between delta power and response inhibition is more evident from childhood to mid-adolescence than at older ages. One possible explanation is that the functional significance of delta activity changes across development. Low-frequency EEG activity, particularly delta and theta power, generally decreases with age across childhood and adolescence (Anderson and Perone, 2018, Meng et al., 2022, Miskovic et al., 2015), and this reduction is widely regarded as a marker of neural maturation, reflecting changes in cortical functional organization and increasing large-scale network integration (Chung et al., 2022, van Noordt and Willoughby, 2021). At younger ages, higher delta power may therefore reflect a relatively less mature neural state characterized by greater low-frequency dominance. Because response inhibition is also still undergoing substantial development during this period (Luna et al., 2004, Williams et al., 1999), individual differences in delta power may be more closely tied to variation in inhibitory performance. By late adolescence, however, individual differences in response inhibition may depend less on a single low-frequency spectral feature and more on the coordinated maturation of the prefrontal cortex and broader control networks (Constantinidis and Luna, 2019, Marek and Dosenbach, 2018, Ojha et al., 2022). From this perspective, the disappearance of the delta effect after 15.20 years of age may indicate that delta power alone is no longer sufficient to capture meaningful variation in response inhibition at later developmental stages.

In the theta band, higher theta power was associated with poorer response inhibition at the bivariate level. However, once age was entered into the model, neither the main effect of theta power nor the theta power × age interaction was significant. This pattern suggests that the observed correlation between theta power and response inhibition may largely reflect their parallel developmental change rather than a stable association between theta power and inhibitory performance per se. Specifically, theta power decreased with age, whereas response inhibition improved with age; once age-related variance was accounted for, theta power no longer explained unique variance in response inhibition. This interpretation is broadly consistent with previous work. Pscherer et al. (2021), for example, found that the association between resting-state theta power and inhibition-related behavior was moderated by age, but was not significant during childhood and adolescence. Pscherer et al. (2022) likewise reported that theta power was associated with neural activity during inhibitory processing, but not directly with behavioral response inhibition. Taken together, these findings suggest that resting-state theta power may be more closely related to developmental background variation or general processing characteristics than to response inhibition itself during childhood and adolescence.

TBR, which reflects the relative balance between theta and beta activity, also did not show a significant interaction with age under the primary HC3-based inference. In the bootstrap sensitivity analysis based on 5000 resamples (Supplementary Table S3), however, the percentile 95% confidence interval for the TBR × age interaction excluded zero, whereas the HC3-based Wald 95% confidence interval included zero. The TBR interaction was therefore considered method-sensitive and was not treated as a robust moderation effect. This pattern differs from that of theta power, for which the interaction was non-significant under both inferential approaches. Conceptually, TBR may capture the relative organization of theta and beta activity and has been associated with broader aspects of executive control. For example, Zhang et al. (2017) linked elevated TBR to atypical interference control, Lin et al. (2021) reported an association between TBR and inhibitory performance in children with ADHD, and Perone et al. (2018) found that TBR remained associated with children’s executive function after controlling for age and verbal ability. Nevertheless, these findings do not establish TBR as a specific marker of response inhibition or support a firm conclusion regarding its age-dependent association with SSRT. We therefore interpret the present TBR result as preliminary and method-sensitive, requiring independent replication before substantive conclusions can be drawn.

In the alpha band, higher alpha power was associated with better response inhibition, and this association remained significant after age was included in the model. However, the alpha power × age interaction was not significant. This pattern suggests that alpha power shows a relatively stable positive association with response inhibition across the age range studied. Previous work points in a similar direction. Whedon et al. (2020) found that increases in frontal alpha power in early childhood predicted better later inhibitory ability, and Wolfe and Bell (2004) reported that preschool children with better Stroop performance showed higher frontal alpha power. From a functional perspective, alpha activity has often been linked to the suppression of irrelevant information and the maintenance of task-relevant processing (Klimesch, 2012). Taken together, the present findings suggest that higher alpha power may reflect a resting-state neural profile that is generally beneficial for response inhibition, and that this association does not vary markedly across childhood and adolescence.

ATR, however, showed a significant age-moderating effect: Higher ATR predicted better response inhibition only before 15.68 years of age. This pattern suggests that, compared with alpha power alone, ATR may be more developmentally sensitive. Resting-state EEG during childhood and adolescence is characterized not only by the maturation of alpha activity, but also by a reduction in theta activity (Anderson and Perone, 2018, Miskovic et al., 2015). Because ATR reflects the balance between alpha and theta activity, it may be more sensitive than alpha power alone to developmental variation in this aspect of spectral organization. Direct evidence linking ATR specifically to response inhibition remains limited, but Cai et al. (2021) found that higher ATR predicted better inhibitory performance in the Receptive Attention Task among children aged 7–9 years, which is broadly consistent with the present finding at younger ages. The disappearance of the ATR effect after 15.68 years of age may indicate that the alpha-theta balance is particularly informative from childhood to mid-adolescence, when individual differences in inhibitory performance are still more closely tied to broad maturational changes in resting-state spectral organization. By late adolescence, however, ATR alone may become less sufficient to capture meaningful variation in response inhibition.

For beta power and BTR, the present study revealed age-dependent reversals in their associations with response inhibition. Higher beta power was associated with better response inhibition before 10.85 years of age but with poorer response inhibition after 14.47 years of age. BTR showed a broadly similar pattern: Higher BTR was associated with better response inhibition before 9.47 years of age but with poorer response inhibition after 14.17 years of age. At younger ages, the positive associations of beta power and BTR with response inhibition may reflect the developmental significance of relatively stronger higher-frequency activity. Resting-state EEG development across childhood and adolescence is generally characterized by decreasing lower-frequency activity and increasing higher-frequency activity. Within this developmental context, higher beta power, or greater beta activity relative to theta, may indicate a comparatively more mature pattern of spectral organization among younger children. Such a pattern may reflect more efficient neural processing and more advanced maturation of the systems supporting cognitive control. This interpretation is broadly consistent with Cai et al. (2021), who found that higher frontal BTR predicted better inhibitory and planning abilities in children aged 7–9 years. At older ages, by contrast, the associations of beta power and BTR with response inhibition became negative, consistent with Ding et al. (2023), who reported that higher beta power was associated with poorer stop-signal performance in healthy young adults. One possible explanation concerns the proposed role of beta activity in maintaining the current cognitive or motor state (Engel and Fries, 2010, Schmidt et al., 2019). Successful performance in the stop-signal task requires the rapid cancellation of an already initiated response. Thus, at later developmental stages, relatively elevated resting beta activity may be associated with a stronger tendency to maintain the current response state, potentially making rapid behavioral updating or stopping more difficult.

These opposing developmental tendencies may also help explain why the behavioral meaning of BTR differs across age. By later adolescence, theta activity has generally declined (Campbell and Feinberg, 2009, Liu et al., 2014) and beta activity has become more prominent (Uhlhaas and Singer, 2010). Individual differences in BTR may therefore increasingly reflect variation in beta power or in the relative predominance of beta over theta. If comparatively high beta dominance is associated with excessive maintenance of the current cognitive or motor state, then a higher BTR may become less favorable for rapid response cancellation. This interpretation also highlights why BTR cannot be regarded as a developmentally invariant marker of cognitive control. Although frontal BTR has been proposed as a potential indicator of attentional control capacity (Putman et al., 2014), its functional meaning may depend on the developmental stage and on the distinct contributions of beta and theta power. The present findings therefore suggest that the association between BTR and response inhibition may reflect the changing significance of the relative beta-theta balance across age rather than a stable effect attributable to either frequency band alone. Nevertheless, because the present study did not formally decompose the respective contributions of beta and theta to the ratio effect, this interpretation should be regarded as provisional. Statistical approaches that jointly model beta, theta, and BTR are needed to clarify their independent and combined developmental contributions.

Compared with task-related EEG, resting-state EEG is generally considered to provide a relatively stable measure of intrinsic neural activity that is less dependent on the demands of a specific task (Naepflin et al., 2007). When considered together, the present findings indicate that several associations between resting-state spectral measures and response inhibition were age-dependent rather than developmentally uniform. First, the associations for delta power and ATR weakened or were no longer statistically significant with increasing age. This pattern suggests that these resting-state spectral characteristics may be more sensitive markers of individual differences in response inhibition during childhood and early adolescence, when neural systems supporting cognitive control are still undergoing substantial reorganization. By later adolescence, as the prefrontal cortex and distributed control networks become more integrated and specialized, individual differences in inhibitory performance may be increasingly shaped by the coordinated functioning of broader neural systems rather than by variation in any single resting-state spectral measure (Constantinidis and Luna, 2019, Marek and Dosenbach, 2018, Ojha et al., 2022). Second, alpha power showed the most developmentally consistent association with response inhibition, with higher alpha power being related to better inhibitory performance across the sampled age range. Rather than indicating a causal protective effect, this stable association may suggest that alpha activity captures a comparatively age-general aspect of neural organization relevant to attentional regulation and inhibitory control. Third, beta power and BTR exhibited a distinct age-dependent reversal (as discussed above), with their associations with response inhibition changing direction as a function of age. Overall, the present findings suggest that resting-state EEG spectral measures should not be interpreted as fixed neural markers with invariant behavioral meaning across development. Rather, their associations with response inhibition appear to depend on age and maturational context, such that resting-state spectral activity is better understood as a developmentally contingent correlate of inhibitory functioning rather than a static indicator whose significance remains constant from childhood through adolescence.

Finally, the upper Johnson–Neyman boundaries for delta power, ATR, beta power, and BTR fell within a relatively narrow mid-adolescent range, from 14.17 to 15.68 years. However, beta power and BTR also showed lower boundaries at 10.85 and 9.47 years, respectively, marking transitions from significant negative associations to non-significant associations before the effects became significantly positive beyond the corresponding upper boundaries. Thus, it was specifically the upper, rather than all, Johnson–Neyman boundaries that converged in mid-adolescence. This convergence may tentatively suggest that some associations between rs-EEG spectral indices and response inhibition become attenuated or change in form during this period. Such an interpretation is broadly compatible with neuroimaging evidence that association cortices, network hubs, and cortico-subcortical connectivity continue to reorganize throughout adolescence, including findings linking brain connectivity around age 14 to subsequent changes in response inhibition (Whitaker et al., 2016, Váša et al., 2020, Wang et al., 2021). Nevertheless, these boundaries are statistical features of the fitted cross-sectional models and should not be interpreted as precise developmental thresholds or evidence of within-individual change. Accordingly, the apparent convergence of the upper boundaries in mid-adolescence should be regarded as an exploratory observation requiring confirmation in longitudinal research.

The present findings have theoretical, practical, and methodological implications. Theoretically, they suggest that the relationship between rs-EEG spectral indices and response inhibition is developmentally dynamic rather than fixed across childhood and adolescence. This perspective may help explain why previous studies have reported inconsistent or even contradictory associations across delta, theta, alpha, and beta bands, as such discrepancies may partly reflect differences in the developmental stages represented in the samples. Practically, the findings may also be relevant to the assessment of response inhibition-related difficulties in children and adolescents. Such difficulties are common in neurodevelopmental disorders such as ADHD and are often associated with impulsivity and poor self-regulation. In this context, rs-EEG spectral indices may provide useful auxiliary neurophysiological information for assessment (Lenartowicz and Loo, 2014, van Hulst et al., 2018). At the same time, the present results emphasize that these indices should not be interpreted without reference to age. Their potential value may depend on being interpreted within an age-referenced developmental framework. Methodologically, the study highlights the importance of examining both single-band power and band-ratio indices. Whereas prior work has focused primarily on single-band measures, the present findings show that single-band and ratio-based indices do not necessarily follow the same developmental pattern. Considering both types of measures may therefore provide a more complete picture of developmental variation in rs-EEG and response inhibition.

Several limitations should be noted. First, because the study used a cross-sectional design, the findings reflect age-related differences between individuals rather than true within-person developmental change and therefore do not support strong causal inference (Kraemer et al., 2000). Accordingly, the age regions identified by the Johnson–Neyman analyses should be interpreted only as statistical boundaries within the current model rather than as precise developmental windows or true turning points. Longitudinal studies are needed to clarify the co-development of rs-EEG spectral indices and response inhibition over time (Foulkes and Blakemore, 2018). Second, chronological age was used as the primary developmental index. Although age is the most commonly used temporal variable in developmental research, it does not fully capture biological maturation, particularly during adolescence, when individuals of the same age may differ substantially in pubertal status, hormone levels, and brain development (Blakemore et al., 2010). Future work could therefore incorporate pubertal or other biological maturation measures to clarify the mechanisms underlying the age-related patterns observed here. Third, the interpretation of band ratio indices should remain cautious. Because band ratios are influenced not only by their component frequency bands but also by broader spectral properties, including aperiodic activity (Donoghue et al., 2020, Finley et al., 2022), the present interpretations should be regarded as preliminary. More refined spectral decomposition methods will be needed to distinguish the functional significance of ATR and BTR more precisely. Finally, the present study primarily relied on linear moderation models. Although this approach provided a parsimonious test of age-related variation, it may not fully capture more complex nonlinear developmental patterns. Future research could use more flexible nonlinear approaches, such as generalized additive mixed models (Wood, 2017) or time-varying effect models (Lanza and Linden-Carmichael, 2021), to further examine whether more complex developmental associations exist.

## 5 Conclusion

This study examined the associations between resting-state EEG spectral indices and response inhibition in children and adolescents aged 6–18 years. The findings indicate that several of these associations vary across age rather than remaining uniform throughout childhood and adolescence. Specifically, age significantly moderated the associations of delta power, beta power, ATR, and BTR with response inhibition, whereas no significant age-moderating effects were observed for theta power, alpha power or TBR. Johnson–Neyman analyses further identified the age ranges within which these associations were statistically significant. Higher beta power and BTR were associated with better response inhibition at younger ages, but with poorer response inhibition at older ages, with neither association reaching significance across the intermediate age range. The positive associations of delta power with poorer response inhibition were no longer significant above approximately 15.20 years of age, whereas the negative association between ATR and SSRT was no longer significant above approximately 15.68 years of age. Together, these results suggest that resting-state EEG spectral indices differ in their age-related associations with response inhibition. They also indicate that band-ratio indices may provide complementary information about the relative balance between frequency components, although their effects should not be interpreted independently of the single bands from which they are derived. Overall, this study provides a developmental perspective on previously inconsistent findings concerning resting-state EEG and response inhibition and underscores the importance of considering age when interpreting the behavioral significance of EEG spectral measures in children and adolescents.

## CRediT authorship contribution statement

**Yanxi Xu:** Writing – original draft, Visualization, Software, Methodology, Investigation, Formal analysis, Data curation. **Ziqi Yang:** Writing – original draft, Visualization, Software, Investigation, Formal analysis, Data curation. **Gaoxiang Ouyang:** Writing – original draft, Software, Methodology, Data curation. **Qinghua He:** Writing – original draft, Software, Methodology, Investigation. **Mengsi Xu:** Resources, Project administration, Methodology, Funding acquisition, Conceptualization.

## Ethics declaration

Written informed consent to take part in the study and to publish the article has been obtained from all participants or their legal representatives. The privacy rights of participants have been observed.

This study was performed in compliance with relevant laws, regulatory frameworks and guidelines where the research took place. This study was approved by the Ethics Committee of the State Key Laboratory of Cognitive Neuroscience and Learning, Beijing Normal University. (Approval No. IRB_A_0054_2023004)

## Ethical standards

This study was performed in line with the principles of the Declaration of Helsinki. Approval was granted by the Ethics Committee of the State Key Laboratory of Cognitive Neuroscience and Learning, Beijing Normal University (No. IRB_A_0054_2023004). Written informed consent was obtained from all participants and their legal guardians.

## Declaration of Competing Interest

The authors declare that they have no known competing financial interests or personal relationships that could have appeared to influence the work reported in this paper.

## Data Availability

Data will be made available on request.
