# Supplementary material for: Developmental variation in resting-state EEG spectral correlates of response inhibition in children and adolescents
Source: Dev Cogn Neurosci. 2026 Oct 6;82:101834. doi: 10.1016/j.dcn.2026.101834 (PMC13676919; doi:10.1016/j.dcn.2026.101834)
Supplement: Supplementary file 1 [file mmc1.docx]

***Table S1****. Comparison of fit indices between linear and quadratic (nonlinear EEG) moderation models for the association between relative power indices and SSRT.*

| Behavior | EEG | model | R^2^ | Adj_R^2^ | AIC | BIC | ∆R^2^ | LRT_p |
| --- | --- | --- | --- | --- | --- | --- | --- | --- |
| SSRT | delta | linear | 0.384 | 0.383 | 4195.060 | 4227.959 | 0.001 | 0.141 |
|  |  | quad | 0.385 | 0.383 | 4195.122 | 4238.988 |  |  |
|  | theta | linear | 0.378 | 0.376 | 4213.392 | 4246.291 | 0.001 | 0.311 |
|  |  | quad | 0.379 | 0.376 | 4215.048 | 4258.914 |  |  |
|  | alpha | linear | 0.386 | 0.385 | 4189.499 | 4222.399 | 0.001 | 0.435 |
|  |  | quad | 0.387 | 0.385 | 4191.828 | 4235.694 |  |  |
|  | beta | linear | 0.384 | 0.383 | 4194.655 | 4227.555 | 0.003 | 0.017 |
|  |  | quad | 0.387 | 0.385 | 4190.450 | 4234.316 |  |  |
|  | ATR | linear | 0.385 | 0.383 | 4193.129 | 4226.029 | < .001 | 0.726 |
|  |  | quad | 0.385 | 0.383 | 4196.486 | 4240.352 |  |  |
|  | BTR | linear | 0.384 | 0.383 | 4194.590 | 4227.489 | 0.001 | 0.203 |
|  |  | quad | 0.385 | 0.383 | 4195.384 | 4239.250 |  |  |
|  | TBR | linear | 0.379 | 0.378 | 4209.710 | 4242.609 | 0.004 | 0.003 |
|  |  | quad | 0.383 | 0.381 | 4201.745 | 4245.611 |  |  |

*Note: The linear model (without quadratic terms) was compared with the quadratic model (including EEG² and EEG²×age) using a nested-model likelihood ratio test (LRT, F-test). Model selection was further evaluated using AIC, BIC, adjusted R², and ΔR². Lower AIC/BIC values indicate better fit balancing parsimony.ΔR² is the increase in R² when adding quadratic terms.*

***Table S2****. Durbin-Watson statistics for linear moderation models.*

| EEG | Durbin_Watson | R^2^ |
| --- | --- | --- |
| delta | 1.856 | 0.383 |
| theta | 1.853 | 0.376 |
| alpha | 1.852 | 0.385 |
| beta | 1.875 | 0.383 |
| ATR | 1.854 | 0.383 |
| BTR | 1.875 | 0.383 |
| TBR | 1.863 | 0.377 |

***Figure S1****. Diagnostic plots for the linear moderation model of delta relative power. Panels show residuals vs. fitted values, Q-Q plot of standardized residuals, and histogram with normal density curve.*

***Figure S2****. Diagnostic plots for the linear moderation model of theta relative power. Panels show residuals vs. fitted values, Q-Q plot of standardized residuals, and histogram with normal density curve.*

***Figure S3****. Diagnostic plots for the linear moderation model of alpha relative power. Panels show residuals vs. fitted values, Q-Q plot of standardized residuals, and histogram with normal density curve.*

***Figure S4****. Diagnostic plots for the linear moderation model of beta relative power. Panels show residuals vs. fitted values, Q-Q plot of standardized residuals, and histogram with normal density curve.*

***Figure S5****. Diagnostic plots for the linear moderation model of TBR. Panels show residuals vs. fitted values, Q-Q plot of standardized residuals, and histogram with normal density curve.*

***Figure S6****. Diagnostic plots for the linear moderation model of ATR. Panels show residuals vs. fitted values, Q-Q plot of standardized residuals, and histogram with normal density curve.*

***Figure S7****. Diagnostic plots for the linear moderation model of BTR. Panels show residuals vs. fitted values, Q-Q plot of standardized residuals, and histogram with normal density curve.*

These plots indicated heteroscedasticity and right-skewness. Accordingly, primary statistical inference was based on HC3 heteroscedasticity-consistent standard errors, corresponding p values, and Wald 95% confidence intervals. Bootstrap percentile 95% confidence intervals based on 5,000 resamples were additionally used as a sensitivity analysis.

***Figure S8****. Cross-sectional age-related distributions of spectral indices and ratios across ages 6-18, stratified by sex.*

***Table S3****. Bootstrap percentile 95% confidence intervals for the focal terms in the moderation models examining the associations between spectral indices and SSRT.*

| **Term** | **LLCI** | **ULCI** |
| --- | --- | --- |
| **delta power** |  |  |
| age | -0.634 | -0.551 |
| delta | 0.046 | 0.121 |
| age × delta | -0.087 | -0.010 |
| **theta power** |  |  |
| age | -0.659 | -0.573 |
| theta | -0.058 | 0.018 |
| age × theta | -0.063 | 0.011 |
| **alpha power** |  |  |
| age | -0.635 | -0.556 |
| alpha | -0.136 | -0.065 |
| age × alpha | -0.011 | 0.067 |
| **beta power** |  |  |
| age | -0.647 | -0.556 |
| beta | -0.077 | 0.016 |
| age × beta | 0.048 | 0.130 |
| **ATR ratio** |  |  |
| age | -0.620 | -0.541 |
| ATR | -0.150 | -0.068 |
| age × ATR | 0.024 | 0.113 |
| **BTR ratio** |  |  |
| age | -0.650 | -0.554 |
| BTR | -0.091 | 0.023 |
| age × BTR | 0.047 | 0.146 |
| **TBR ratio** |  |  |
| age | -0.651 | -0.557 |
| TBR | -0.036 | 0.056 |
| age × TBR | -0.088 | -0.009 |

*Note. The focal terms reported are age, the spectral index, and the age × spectral index interaction; the intercept and sex covariate are omitted for concision. LLCI and ULCI represent the lower and upper limits, respectively, of the bootstrap percentile 95% confidence intervals based on 5,000 resamples. The bootstrap confidence intervals were used as a sensitivity analysis; primary statistical inference was based on HC3 robust standard errors, corresponding p values, and Wald 95% confidence intervals reported in Table 1.*
